# Comprehensive Review: Mavacamten and Aficamten in Hypertrophic Cardiomyopathy

**DOI:** 10.3390/biomedicines13071619

**Published:** 2025-07-01

**Authors:** Helin Savsin, Tomasz Tokarek

**Affiliations:** 1 Center for Innovative Medical Education, Jagiellonian University Medical College, Medyczna 9, 31-008 Krakow, Poland; hsavsin1@gmail.com; 2 Center for Invasive Cardiology, Electrotherapy and Angiology, Kilinskiego 68, 33-300 Nowy Sacz, Poland

**Keywords:** mavacamten, aficamten, cardiomyopathy, cardiac myosin inhibitors, disease-modifying therapy, left ventricular hypertrophy

## Abstract

Hypertrophic cardiomyopathy (HCM) is the most common monogenic heart disease, with an estimated prevalence of 1:600 in the general population, and is associated with significant morbidity. HCM is characterized by left ventricular hypertrophy and interventricular septal thickening due to sarcomere protein gene mutations. The recent emergence of cardiac myosin inhibitors (CMIs), specifically mavacamten and aficamten, has introduced a paradigm shift in HCM management by directly targeting the hypercontractile state of the disease. This review comprehensively discusses the molecular mechanisms of mavacamten and aficamten, highlighting their biochemical similarities and differences from available data. It evaluates their reported efficacy in completed clinical trials, such as reducing left ventricular outflow tract (LVOT) obstruction, improving functional capacity, and enhancing quality of life in HCM. It further provides insight and updates to ongoing trials of both CMIs. Finally, it compares and elaborates on the safety profiles of mavacamten and aficamten, discussing their favorable safety profiles shown in completed studies. In current clinical practice, only mavacamten is approved for use, and clinical insights concerning both CMIs are limited, but encouraging. In summary, cardiac myosin inhibitors are a promising class of disease-modifying drugs for HCM with proven short-term safety and efficacy, but limited data are available to fully determine their long-term effects and efficacy in diverse patient populations. Ongoing research is necessary to further explore and define their role in HCM management.

## 1. Introduction

**Figure 1:**
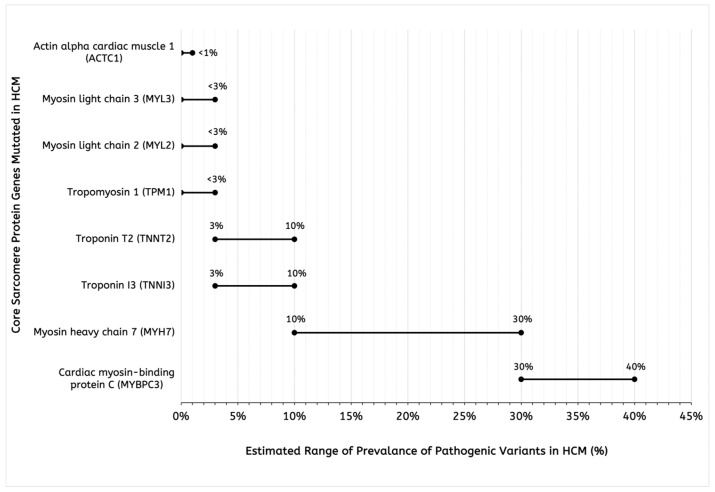
Estimated ranges of prevalence of pathogenic variants in core sarcomere genes in hypertrophic cardiomyopathy (HCM). The data were taken from the Genome Aggregation Database derived from a large-scale meta-analysis of genetic cohort data [11]. The genes are displayed in order of prevalence.

Hypertrophic cardiomyopathy (HCM) is a prevalent autosomal-dominant cardiac myocyte disorder caused by sarcomere protein gene mutations leading to abnormal thickening of the heart muscle, particularly the interventricular septum [1]. In multiple studies, HCM had an estimated prevalence of 1:652–1:344 individuals in the general adult population [2,3,4,5]. Although HCM is inherited in an autosomal-dominant manner, it has variable expressivity and penetrance [6]. This signifies that patients with HCM may have ranging disease severity and onset, with individuals not having to carry a pathogenic variant to present with the phenotypic features of HCM. To date, HCM has been linked to over 900 individual mutations present in more than 20 genes [7], the most common mutations being in the β-cardiac myosin heavy chain, including the myosin heavy chain 7 (MYH7) gene and myosin-binding protein C3 (MYBPC3) gene, which were present in 70% of variant positive patients [8]. Importantly, they are part of the eight total sarcomere genes that are determined to definitively cause HCM (Figure 1). A majority of mutations in HCM affect genes that encode for thin or thick myofilament proteins within the cardiac sarcomere. Despite this, the genetic etiology of many HCM patients remains unclear. A small group of these patients also carry non-sarcomere gene mutations and still present with the typical symptoms and signs of HCM [9]. It is not entirely understood how sarcomere variants lead to the clinical phenotype of HCM. In clinical practice, the European Society of Cardiology guidelines of HCM management suggest genetic testing and counseling of newly diagnosed patients (Class IB recommendation) to aid in decision-making and setting up regular screening of families with inherited HCM [10].

HCM is broadly categorized into obstructive and non-obstructive types, each with different prognosis and management. Obstructive HCM (oHCM) is characterized by left ventricular outflow tract (LVOT) obstruction at rest or exertion due to the highly contracted myocardium [12]. These patients present with a high overall risk of advanced heart failure and atrial fibrillation [13]. HCM can also result in impaired diastolic filling, obstruction of LVOT, arrhythmias, and an increased risk of sudden cardiac death [14]. Conversely, in non-obstructive HCM (nHCM), patients lack the obstructive pathophysiology of the disease and are mainly considered to be asymptomatic or only have mild symptoms [12].

The majority of HCM patients are asymptomatic or mildly symptomatic and therefore do not require treatment. For symptomatic patients with HCM, the treatment is focused on symptom management through beta-blockers. This class of drugs decrease ischemic chest discomfort and may reduce LVOT obstruction and resultant dyspnea during exercise due to their negative chronotropic and inotropic effects [15]. Another cornerstone treatment is L-type calcium channel blockers, which are alternatives to beta-blockers in patients that are unresponsive to a previous course of treatment [16]. These drugs mainly include verapamil or diltiazem due to their improvement of ventricular diastolic filling by reducing temporal asynchrony [17]. Additionally, diuretics may also be used to treat heart failure and decrease pulmonary congestion in HCM. The use of diuretics requires continuous monitoring to avoid hypotension, hypovolemia, or onset of LVOT obstruction in HCM patients [17].

In terms of surgical treatment, septal myectomy is the primary approach used in obstructive HCM due to the effective symptom relief caused by reduced outflow tract obstruction [18]. It consists of removing hypertrophied septal muscle to reduce LVOT gradient in symptomatic patients with refractory medical treatment [18]. Another minimally invasive intervention is percutaneous transluminal septal myocardial ablation, also known as alcohol septal ablation (ASA). During the procedure, ethanol is injected percutaneously into the first or second septal perforation of the left anterior descending artery, which induces an iatrogenic myocardial infarction throughout the basal septum [19]. This initiates long-term remodeling to reduce obstruction of the LVOT [20]. Throughout the last fifty years, there has been an increase in different surgical techniques and pharmaceutical treatments used in HCM due to the dramatic improvement in the understanding of the disease entity.

Despite maximal medical intervention and therapy, many HCM patients still demonstrate high cumulate burden directed by long-term heart failure and atrial fibrillation [21]. Furthermore, it has previously been discussed in an analysis of the Sarcomeric Human Cardiomyopathy Registry that young age and the presence of sarcomere mutations are strong predictors of adverse outcomes in these patients [21]. A large, multicenter cohort study from 2020 reported significant excess lifetime mortality in HCM patients [22]. The persistent burden of HCM, especially on young patients, shows an urgent unmet need for disease-modifying treatment that alter the underlying muscle pathophysiology.

Cardiac myocyte inhibitors (CMIs) are a novel group of drugs inhibiting the myosin ATPase, inducing a return to a normal or quasi-normal state of cardiac contractility [1]. Currently, mavacamten and aficamten are the two main CMIs undergoing clinical trials. Mavacamten is a novel allosteric modulator of cardiac myosin, offering a new therapeutic approach by directly targeting the hypercontractile state of HCM [1]. Aficamten, an even newer CMI, is also an allosteric inhibitor of myosin; however, it binds to a distinct allosteric site on the catalytic domain of the protein [23].

This review discusses the key features of two main CMIs, mavacamten and aficamten, their differing mechanisms of action, recently completed trials, ongoing trials, and safety profiles, and identifies future directions of the application of CMIs in different clinical settings to treat HCM.

## 2. Mechanism of Action

**Figure 2:**
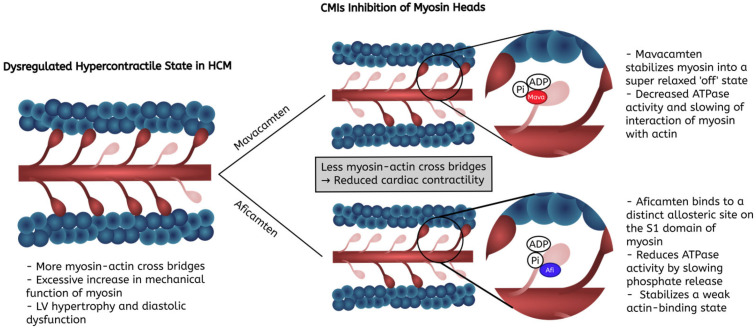
Mechanisms of action of mavacamten and aficamten, showing their differing binding sites on the cardiac myosin in hypertrophic cardiomyopathy. The figure illustrates how mavacamten (“Mava” in bright red) and aficamten (“Afi” in bright blue) change the activity of myosin heads. Mavacamten binds to catalytic domains of the myosin S1 head, stabilizing an “off” state by decreasing available myosin heads for actin binding. Aficamten binds to a distinct allosteric site from mavacamten, specifically slowing phosphate release in return reducing crossbridge binding. Both agents through differing mechanisms reduce cardiac hypercontractility in hypertrophic cardiomyopathy. Abbreviations: HCM: hypertrophic cardiomyopathy; LV: left ventricle.

In HCM, excessive myosin-actin crossbridges are present, inducing the hallmark dysregulated hypercontractile state of the disease [24]. Mavacamten operates by binding to the catalytic domain of cardiac myosin, thereby inhibiting its ATPase activity. This inhibition reduces the number of myosin heads interacting with actin filaments, leading to fewer crossbridges formed during the cardiac cycle (Figure 2) [25]. Crystallographic and biochemical studies have shown that mavacamten “locks” the myosin in a state that is less prone to participate in the contractile cycle [26]. Consequently, mavacamten decreases myocardial contractility, alleviates LVOT obstruction, and improves myocardial energetics and diastolic function [27]. By directly modulating the contractile apparatus of the heart, mavacamten addresses the fundamental hyperdynamic state seen in HCM.

Similarly, aficamten is a direct inhibitor of the cardiac myosin S1 subfragment of the myosin catalytic domain [28], resulting in a change in myosin conformation that strongly decreases the rate of ATP turnover and thus reducing cardiac contractility [23]. While sharing the goal of reducing myosin-mediated hypercontractility, aficamten has a distinct allosteric site on the myosin head and has a different phenotype of phosphate release, slowing its release to a greater extent in comparison to mavacamten (Figure 2) [23]. According to recent structural and molecular data, aficamten is a more potent stabilizer of phosphate release caused by actin activity with very low ATP turnover rates in comparison to mavacamten [23]. As a result, aficamten has a different dose–response profile with a shallower relationship between dosage and reductions in LVEF [23]. Therefore, aficamten has a more rapid onset and more rapid reversibility of its inhibition of myosin, contributing to its shorter half-life (Table 1). These structural differences can possibly allow different clinical advantages such as more immediate dose titration in the case of aficamten, which is under investigation in clinical trials currently.

## 3. Clinical Efficacy

### 3.1. Mavacamten Clinical Trials

#### 3.1.1. Phase II Trials: PIONEER-HCM

The PIONEER-HCM (Phase II Open-Label Pilot Study Evaluating Mavacamten in Subjects with Symptomatic Hypertrophic Cardiomyopathy and Left Ventricular Outflow Tract Obstruction) trial was a foundational study measuring the impact of mavacamten in patients with symptomatic obstructive HCM. This multi-center, open-label trial included patients with significant LVOT obstruction despite optimal medical therapy. Patients were divided into two main cohorts to evaluate mavacamten’s effects as monotherapy versus an add-on therapy. In cohort A, patients received 10–20 mg/day of mavacamten as monotherapy without other medications, while in cohort B, patients had a combination of mavacamten 2–5 mg/d with beta-blockers [29]. The primary endpoint was the change in LVOT gradient from baseline to week 12, while secondary endpoints included changes in NYHA functional class, exercise capacity (peak oxygen consumption, VO_2_ max), and biomarkers of myocardial stress and injury.

Results from PIONEER-HCM were encouraging. Mavacamten significantly reduced the postexercise LVOT gradient in cohort A from 103 mm Hg (SD, 50) at baseline to 19 mmHg (SD, 13) at 12 weeks (mean change, −89.5 mmHg [95% CI, −138.3 to −40.7 mmHg]; *p* = 0.008), meeting its primary endpoint [29]. Similarly, within the same cohort, a reduction in LVEF with a mean change of −15% [CI, −23% to −6%] and an increase in peak VO_2_ by a mean of 3.5 mL/kg/min (CI, 1.2 to 5.9 mL/kg/min) was observed [29].

Cohort B also met the primary endpoint by demonstrating a reduction of postexercise LVOT gradient from 86 mmHg (SD, 43) to 64 mmHg (SD, 26) (mean change, −25.0 mmHg [CI, −47.1 to −3.0 mmHg]; *p* = 0.02), and mean change in resting LVEF of −6% (CI, −10% to −1%). Within the same cohort, an increase in peak VO_2_ by a mean of 1.7 mL/kg/min (SD, 2.3) (CI, 0.03 to 3.3 mL/kg/min) was observed [29]. Further secondary endpoints such as dyspnea, NT-proBNP, and troponin were improved in both cohorts, while many patients also showed NYHA functional class improvement as they changed from class III to class I or II [29]. These changes indicate a decrease in myocardial stress and injury.

#### 3.1.2. Phase II Trials: MAVERICK-HCM

The MAVERICK-HCM study was a double-blind, placebo-controlled, randomized study to determine mavacamten’s effects in non-obstructive hypertrophic cardiomyopathy (nHCM) patients. The inclusion criteria qualified patients with symptomatic nHCM (with NYHA class II–III), elevated NT-proBNP levels (≥300 pg/mL), and preserved ejection fraction (LVEF ≥ 55%). Unlike prior trials focused on obstructive mechanisms, MAVERICK-HCM explored mavacamten’s role in a cohort without LVOT obstruction. The primary objective was tolerability and safety, while secondary endpoints included changes in biomarkers such as NT-proBNP and high-sensitivity troponin I, as well as exploratory endpoints like ECG parameters and burden of disease.

Mavacamten was found to be well tolerated with mostly mild to moderate adverse effects in these patients. Serious side effects occurred in 10% of participants on mavacamten and 21% on placebo [30]. Most serious adverse events were cardiovascular such as recurrent atrial fibrillation or atrial flutter. Overall, 5 participants out of the 59 met the stopping criteria due to a documented LVEF < 45%, and all 5 of these participants recovered with their LVEF values increasing towards baseline after 4–12 weeks [30].

Notably, mavacamten led to significant reductions in NT-proBNP, with mavacamten treated groups experiencing a 53% decrease in the geometric mean compared to a 1% decrease in the placebo group [30]. This corresponds to a geometric mean difference of −435 pg/mL for mavacamten versus −6 pg/mL for placebo (*p* = 0.0005) [30]. Similarly, the geometric mean of cTnI levels was also reduced 34% in the mavacamten group, in contrast to 4% in the placebo group [30]. The geometric mean differences were −0.008 ng/mL for mavacamten and 0.001 ng/mL for placebo (*p* = 0.009) [30]. These results generally suggest a decrease in myocardial wall stress and injury. While the trial was not designed for clinical efficacy outcomes, exploratory analyses showed improved symptom burden and stabilization or modest improvement in diastolic function. MAVERICK-HCM was the first controlled trial to prove mavacamten’s potential in nHCM patient populations, supporting the disease-modifying effects beyond gradient reduction, as observed in other studies.

#### 3.1.3. Phase III Trials: EXPLORER-HCM

The EXPLORER-HCM trial, a larger and more rigorous randomized, double-blind, placebo-controlled study, was built upon the PIONEER-HCM findings. This trial included a diverse patient population and extended follow-up period. The primary endpoint was the proportion of patients achieving a ≥1.5 mL/kg/min increase in peak VO_2_ and a ≥1 NYHA class improvement or a ≥3.0 mL/kg/min increase in peak VO_2_ without NYHA class worsening at week 30. Secondary endpoints were changes in post-exercise LVOT gradient, pVO2, NYHA class, including two scores of functional status: Kansas City Cardiomyopathy Questionnaire-Clinical Summary Score (KCCQ-CSS), and Hypertrophic Cardiomyopathy Symptom Questionnaire Shortness-of-Breath sub-score (HCMSQ-SoB) [31].

EXPLORER-HCM met its primary endpoint, with significantly more patients in the mavacamten group (45 [37%] of 123 patients) achieving the composite outcome compared to the placebo group (22 [17%] of 128 patients) with a difference of +19.4%, 95% CI 8.7 to 30.1; *p* = 0.0005) [31]. Mavacamten-treated patients also showed significant improvements in peak VO_2_ (+1.4 mL/kg per min, 0.6 to 2.1; *p* = 0.0006), NYHA functional class, and LVOT gradient (−36 mm Hg, 95% CI −43.2 to −28.1; *p* = 0.0001) [31]. Moreover, these patients reported substantial enhancements in quality of life, as measured by the KCCQ-CSS and HCMSQ-SoB (KCCQ-CSS +9.1, 5.5 to 12.7; HCMSQ-SoB −1.8, −2.4 to −1.2; *p* = 0.0001) [31]. The study concluded that mavacamten treatment led to significant improvements in exercise capacity, LVOT obstruction, NYHA class and general health status in oHCM patients. The main findings from clinical trials of mavacamten are summarized in Table 2.

### 3.2. Aficamten Clinical Trials

#### 3.2.1. Phase III Trials: SEQUOIA-HCM

The SEQUOIA-HCM trial was a phase III, randomized, placebo controlled, double-blind trial that specifically measured the safety and efficacy of aficamten in symptomatic obstructive hypertrophic cardiomyopathy patients. In total, 282 patients (aficamten = 142, placebo = 140) were enrolled, with inclusion criteria requiring patients to be aged 18 to 85 years with confirmed HCM, left ventricular wall thickness of at least 15 mm, and left ventricular ejection fraction (LVEF) of at least 60% [32]. Randomized patients were assigned either placebo or aficamten over 24 weeks with a following 4-week washout period. The dosages of aficamten were set to start at 5 mg with potential incremental increase up to 20 mg, guided by echocardiographic assessments of LVEF and LVOT gradients.

The primary endpoint was change in peak oxygen uptake VO_2_ during exercise from baseline to week 24. The results of the study showed significant improvement in the patients with aficamten compared to placebo, with a mean difference of 1.7 mL/kg/min (*p* = 0.0001) [32]. Secondary endpoints included a change from baseline to week 12/24 of Kansas City Cardiomyopathy Questionnaire Clinical Summary Score (KCCQ-CSS), NYHA functional class, and LVOT gradient after Valsalva to measure the further efficacy of aficamten. At the end of the 24 weeks, the patients showed improvements on the KCCQ-CSS, with a mean increase of 12 points vs. 5 points in the placebo group (*p* = 0.001) [32]. In the aficamten-treated patient population, 49.3% were able to keep their LVOT gradient < 30 mmHg after Valsalva at week 24 compared to 3.6% in the placebo cohort (*p* = 0.001) [32]. The benefit of aficamten was further supported through improvement in secondary outcomes of this study. Likewise, the favorable safety profile was also demonstrated by the serious adverse events present in only 5.6% of patients versus 9.3% in the placebo group [32]. These findings illustrate the utility of aficamten in symptomatic HCM management, providing improvements in exercise capacity, functional tests, and symptom burden.

#### 3.2.2. Phase II Trials: REDWOOD-HCM

REDWOOD-HCM was an open-label, phase II trial that evaluated the safety and efficacy of aficamten in patients with symptomatic nonobstructive hypertrophic cardiomyopathy characterized by a left ventricular outflow tract gradient ≤ 30 mmHg, LVEF ≥ 60%, and NT-proBNP > 300 pg/mL [33]. Patients were given titrated doses of aficamten based on echocardiographic LVEF over 10 weeks. At week 10, 55% of patients showed an improvement of ≥ 1 New York Heart Association class, and 11 (29%) became asymptomatic [33].

In results, clinically relevant improvements were seen in 55% of patients with reductions in NT-proBNP (56%; *p* = 0.001) and high-sensitivity troponin I (22%; *p* = 0.005) [33]. Average LVEF declined by 5.4% and three asymptomatic patients experienced LVEF < 50%, but they all recovered after the washout period [33]. One participant with prior aborted sudden cardiac death experienced a fatal arrhythmia [33]. Overall, aficamten was generally well tolerated and led to improvements in symptoms and cardiac biomarkers. The main findings from clinical trials of aficamten are summarized in Table 3.

## 4. Ongoing Trials

Mavacamten is currently approved for use in symptomatic obstructive HCM, but its efficacy is still debated in nonobstructive HCM. Mavacamten in Non-Obstructive Hypertrophic Cardiomyopathy (ODYSSEY-HCM, NCT05582395) is a randomized, phase III, double-blind, placebo-controlled clinical trial measuring the safety, tolerability, and efficacy of mavacamten in symptomatic non-obstructive hypertrophic cardiomyopathy (nHCM) [34]. This study will assess improvements at week 48 with two primary endpoints (1) Kansas City Cardiomyopathy Questionnaire 23-item Clinical Summary Score; and (2) peak oxygen consumption (pVO2) on cardiopulmonary exercise testing [34]. In addition, in Part C of the trial, a long-term follow-up to 156 weeks with a once-daily dose of mavacamten, will be conducted for continued assessment of the endpoints and long-term safety. In April 2025, Bristol Myers Squib announced that ODYSSEY-HCM did not meet its dual primary endpoints (KCCQ-23-CSS score and peak oxygen consumption measures) of changes from baseline to week 48 compared to the placebo group [35]. This preliminary statement shows that obstructive and non-obstructive HCM may require distinct treatments tailored to specific pathophysiological mechanisms. The efficacy proven in randomized controlled trials in obstructive HCM patients may not be reflected in non-obstructive HCM patients. This underlines the need for dedicated research to establish evidence-based interventions in nHCM, where a significant need remains for novel disease-modifying treatments.

For aficamten, there is currently an active phase III, placebo controlled clinical trial, the MAPLE-HCM (Metoprolol vs. Aficamten in Patients with LVOT Obstruction on Exercise Capacity in HCM; NCT05767346) study, in which the efficacy of aficamten versus metoprolol as a first-line therapy in oHCM will be measured [36]. This study is ongoing, with the aim of exploring the potential position of aficamten as a superior first-line treatment option. In May 2025, Cytokinetics released positive topline results from MAPLE-HCM through a press release. In this preliminary statement, it was stated that MAPLE-HCM met its primary endpoint of aficamten reaching improvement in pVO_2_ at Week 24, proving its superiority to metoprolol with a favorable safety and tolerability profile [37]. This is one of the initial indications of aficamten’s safety and efficacy, making it a possible monotherapy option in patients with obstructive symptomatic HCM.

Another ongoing clinical trial of aficamten, ACACIA-HCM is building on the encouraging findings from cohort 4 of REDWOOD-HCM, the phase II clinical trial that showed that aficamten resulted in statistically significant improvements in heart failure symptoms and cardiac biomarkers in patients with non-obstructive HCM [33]. ACACIA-HCM is a phase III, multi-center, randomized, double-blind, placebo controlled clinical trial to evaluate the effect of aficamten compared to placebo on health-related quality of life in participants with symptomatic nHCM [38]. This trial is investigating the therapeutic use of aficamten beyond obstructive HCM.

Currently, the ongoing FOREST-HCM extension study (NCT04848506) aims to provide insight into the long-term use of aficamten with previous aficamten trial participants with HCM [39]. This study aims to provide data on durability and long-term safety, and the results are expected to be published in 2028.

On the other hand, CEDAR-HCM is a multi-center, randomized, double-blind, placebo-controlled, open-label extension clinical trial of aficamten in symptomatic oHCM in a pediatric population. It is currently open to enrollment, and it aims to assess the efficacy and safety of aficamten in the pediatric HCM population [40]. The objective of this trial is to fill the unmet treatment needs of HCM as a genetic disease in pediatric populations.

## 5. Safety Profile

Mavacamten has been generally well tolerated in randomized and open-label clinical trials [25,29,31]. Common adverse effects include dizziness, hypotension, and syncope, primarily due to its negative inotropic effects (more listed in Table 4). In the PIONEER-HCM and EXPLORER-HCM trials, these adverse events were manageable with dose adjustments and monitoring. Importantly, no patients in the mavacamten group experienced treatment-related serious adverse events leading to discontinuation.

While long-term safety data are still being collected, interim analyses suggest a favorable risk–benefit profile. Regular echocardiographic monitoring is recommended to assess left ventricular function and avoid potential heart failure due to excessive myocardial relaxation. Mavacamten is currently approved by numerous regulatory agencies such as the FDA (Food and Drug Administration) and EMA (European Medicines Agency) for oHCM treatment; however, due to the aforementioned adverse effects such as heart failure risk, interactions with drugs, and reduced LVEF, the use of the drug is tightly regulated [42].

The mechanism of action of CMIs works to decrease the pathological hypercontractility in patients with HCM, thereby causing a modest reduction in LVEF [43]. Mavacamten is set to require tight regulation of plasma concentration due to its inconsistent pharmacokinetic–pharmacodynamic relationship. In the context of these clinical trials, pharmacokinetics refers to the dosage of these drugs. In the case of mavacamten, the dosage sometimes remained high, such as in PIONEER-HCM, where mavacamten plasma concentrations ≥ 1000 ng/mL were associated with increased reductions in LVEF beyond what was required to eliminate the LVOT gradient [29]. Furthermore, the risk of LVEF < 50% and heart failure on mavacamten was low in the first few months of initiation of the drug, and the risk increased over time due to the prolonged exposure time and potential accumulation of mavacamten concentrations [41]. Due to these results, the EMA recommends genotyping of CYP2C19 to predict poor metabolizers that might show higher maximum concentrations and areas under the curve after a single dose of mavacamten compared with normal metabolizers, which increases the risk of adverse effects [41,44]. Due to these limitations, mavacamten remains a second-line treatment option for symptomatic oHCM patients who are not responsive to beta-blockers or calcium channel blockers [14]. For nHCM, mavacamten is not currently approved for use.

In contrast to mavacamten, aficamten is shown to have less adverse effects in comparison with the early data (Table 4). Based on the review of the 10 clinical trials assessed, the population on mavacamten experienced higher rates of heart failure, atrial fibrillation and treatment interruption originating from a decrease of LVEF < 50% compared to aficamten [41]. This, however, is a limited analysis, as aficamten trials are shorter in general. When it comes to the pharmacodynamic relationship, aficamten shows a more consistent linear drug level to LVEF change and metabolic pathways with lower DDI potential, confirmed by multiple trials [33,41,45,46,47]. Due to these characteristics, aficamten does not require any metabolizer genotyping or medication review, thereby making the prescription of these drugs more convenient for doctors [41,45,46]. In various studies, aficamten was shown to have a shorter half-life (3.5 days), which allows more rapid uptitration of the study drug [32,41]. This gives aficamten the potential to be titrated more rapidly, safely, and in a controlled manner. In comparison to placebo, adverse events associated with treatment, such as dizziness, syncope, and heart failure, appeared to occur at higher rates on mavacamten compared with aficamten among the clinical trials [41]. However, longer follow-up data from long-term extension studies are needed to further examine these effects.

Overall, CMIs are currently considered safe and efficient due to reassuring safety analysis in clinical trials lasting from 10–30 weeks in over 400 patients who completed phase II and phase III trials since 2016 [48]. However, long-term safety is not determined at scale. Currently, the use of mavacamten in clinical practice is complex and intricate for physicians. The regulations are designed to ensure the safe use of the drug following the clinical trials’ findings [41,49]. As of now, there are no risk evaluation, mitigation, or drug monitoring strategies for aficamten, as it is not yet approved for use in HCM.

Despite these analyses, both mavacamten and aficamten have been shown to be safe drugs on short-term analysis and are beneficial when it comes to reducing symptom burden, cardiac biomarkers, and reducing LVOT obstruction with symptomatic oHCM trial patients improving in exercise capacity. Once both these drugs are commercially available, a comparison of mavacamten and aficamten in oHCM could further our understanding of the safety of these medications, without the limitations of study protocols.

## 6. Clinical Insights

Current clinical guidelines solely support mavacamten, the first-in-class cardiac myosin inhibitor, due to its approval by the FDA and EMA. In the 2024 American Heart Association (AHA) and American College of Cardiology (ACC) guidelines, mavacamten was recommended as a possible option in adult symptomatic oHCM patients (usually within NYHA functional class II or III) that were not tolerant or responsive to first-line therapies such as beta-blockers or non-dihydropyridine calcium channel blockers (Class 1B-R recommendation) [14]. This recommendation is especially useful in centers that do not have access to interventional cardiologists or surgeons to offer patients septal reduction therapy. Non-obstructive HCM patients should not be treated with mavacamten outside of research purposes in clinical trials. Within the mavacamten-treated patients, routine monitoring with echocardiography is required to monitor the LVEF. The AHA–ACC guidelines emphasize that patients who develop a LVEF < 50% should be dose-reduced, and if systolic function does not improve, the drug should be discontinued regardless of the presentation of any adverse events or symptom reduction (Class 1 recommendation) [14].

In terms of mavacamten’s potential as an add-on therapy, the promising results of PIONEER-HCM cohort B has prompted the medical community to start implementing mavacamten as add-on therapy to beta-blockers [29]. This is further integrated into a clinical context by the European Society of Cardiology guidelines. In the 2023 ESC guidelines, it is suggested that mavacamten may be combined with beta-blockers or calcium antagonists in an effort to improve LVOT obstruction (Class IIa, A) [10]. It is also possible to use mavacamten as monotherapy if patients have contraindications to the adjunctive first-line therapy options mentioned earlier. A special consideration in clinical practice included pregnant women with oHCM. In the guidelines, expert consensus states that mavacamten should be avoided during pregnancy due to its potential teratogenic effects such as fetal toxicity [10].

Although guidelines do not include aficamten for obstructive HCM treatment yet, the Food and Drug Administration is currently reviewing its New Drug Application, with an expected regulatory decision by 26 December 2025 [50]. However, according to recent data from SEQUOIA-HCM and MAPLE-HCM trials, it could potentially be a more pharmacokinetically favorable option, as previously discussed in the Safety Profile section [32,37]. A potential distinction in the future clinical use of these two CMIs concerns their clinical positioning. Data from cohort B in PIONEER-HCM demonstrated that the combination of mavacamten and beta-blockers has superior outcome results in comparison to monotherapy with mavacamten in oHCM patients [29]. Therefore, in current clinical practice, most physicians use mavacamten as an add-on therapy with other first-line treatments like beta-blockers as per the guideline recommendations. On the contrary, preliminary results from MAPLE-HCM point to aficamten’s superiority in comparison to beta-blockers, as it achieved its primary endpoint of improving peak exercise capacity [37]. These results suggest that aficamten may be more effective as standalone therapy in oHCM patients in contrast to mavacamten which is currently mostly used in combination with other first-line options.

In a large meta-analysis of the previously mentioned RCTs of mavacamten and aficamten (n = 826), cardiac myosin inhibitors were associated with lowered LVOT gradients (with an average reduction of −57.3 mmHg) and decreased LVEF (with an average reduction of −4.74%) [51]. There were substantial improvements in functional parameters like NYHA improvement (RR 2.21) and KCCW-CSS (with an average of +7.71 points) and decreased biomarkers like NT-pro BNP and cardiac troponin I (cTnI) [51] In one real-world cohort study with mavacamten treatment from John’s Hopkins HCM center, 60%–70% of patients improved at least one NYHA class with LVOT gradient reductions that were higher than reported RCT data (reduction of approximately 70 mmHg in comparison to 45 mmHg in EXPLORER HCM) [52]. These findings support that within clinical practice, CMI treatment can translate to patients improving functionally and symptomatically with decreased chest pain and dyspnea and increased exercise tolerance and overall quality of life.

Cardiac troponin I (cTnI) is a recognized predictor of myocardial injury in HCM patients. Approximately 22%–74% of HCM patients have elevated cardiac troponins, which are associated with disease severity, much like the LVOT gradient [53]. During clinical trials, it was discovered that CMIs, especially mavacamten, could decrease cTnI concentrations. For example, in the previously discussed EXPLORER-HCM trial, the mavacamten-treated group showed a significant reduction of cTnI levels over the 30-week period [31]. Similarly, in the secondary endpoint analysis of the MAVERICK-HCM study, it was shown that mavacamten group had a 34% decrease in cTnI in comparison to a mere 4% in the placebo group (*p* = 0.009) [30]. In contrast to mavacamten, clinical trials of aficamten show mainly insignificant reductions in cTnI. In the REDWOOD-HCM trial, aficamten-treated cohorts 1 and 2 showed an insignificant reductions of 18% (*p* = 0.29) and 26% (*p*  =  0.097) of cardiac troponin when compared to the placebo group [53].

Across multiple RCTs, therapeutically induced troponin reductions were reversible once the CMIs were discontinued (post-washout period), which may indicate direct pharmacological influence of improved diastolic function [53,54]. Although not all studies showed statistically significant reductions in troponin levels, monitoring cardiac troponin could be a strong prognostic factor, as it points to improved contractility in HCM patients. Furthermore, it may be an indicator of therapeutic efficacy or adherence to CMI therapy clinically, particularly in mavacamten use. Further research is required to determine the clinical utility of cTnI in response to CMI therapy in HCM.

## 7. Future Directions

CMIs, due to their precise and targeted therapeutic action, are potentially applicable to other cardiac conditions characterized by hypercontractility and diastolic dysfunction. Ongoing research is exploring mavacamten’s use in conditions beyond HCM, such as heart failure with preserved ejection fraction (HFpEF) [55]. Preliminary data from these studies are promising, suggesting that mavacamten could become a versatile therapeutic agent in cardiology [55].

Another big gap in CMIs is the long-term efficacy and safety of the drugs. There is still limited long-term follow-up on aficamten and mavacamten, particularly regarding sustained efficacy, side effects, and disease regression. It is also important to monitor any late-emerging side effects or adverse cardiac remodeling in the group of patients that have been utilizing CMIs. This future data are required to integrate CMIs as long-term treatment strategies for HCM.

CMIs are commonly discussed in symptomatic oHCM, and there are currently no definitive data or conclusion concerning their clinical benefits in nHCM. However, there are ongoing trials exploring this possibility, such as ACACIA-HCM for aficamten and ODYSSEY-HCM for mavacamten [35,38]. Unlike obstructive HCM, where therapies target left ventricular outflow tract obstructions, nHCM lacks in specific treatment options. Patients who suffer from nHCM have to be managed through symptomatic relief using beta-blockers and CCBs without any disease-modifying therapies. A potential new therapy such as CMIs in this field could prevent disease progression including development of HF or arrhythmias.

Additionally, these drugs may have varying effects across different patient populations, including potential sex-specific differences. The impact of CMIs and their treatment outcomes in women are under-researched, especially in the real-world phase after the drug’s approval [56]. A post hoc analysis of sex from EXPLORER-HCM trial of mavacamten reported that women with symptomatic oHCM were older, more likely to be in NYHA class III, with higher NT-proBNP levels [57]. Despite these baseline differences, women treated with mavacamten had similar improvements in primary and secondary endpoints, with greater improvement in NT-proBNP levels after 20 weeks [57] These results suggest that women may have greater treatment gains compared to the male population. Future research should explore potential sex-specific differences to cardiac myosin inhibitors with consideration to clinical profiles and outcome designs for women with HCM.

To advance the therapeutic development of CMIs, future research should also explore the efficacy of cardiac myosin inhibitors in genotypically different populations. HCM is generally regarded as a sarcomere disease with mutations in genes such as MYH7 and MYBPC3, as they cause the dysfunction in the sarcomere leading to hypercontractility [58]. This mechanism of action is the primary rationale for inhibition of the myosin heads with CMIs, which were proven effective in the trials discussed previously. Currently, the drug response to CMIs in HCM patients with non-sarcomere mutations remains unclear. While genotype is mostly collected in clinical trials, HCM continues to be grouped phenotypically as obstructive or non-obstructive to guide clinical decisions. A study testing mavacamten (MYK−461) on in vitro gene-edited human induced pluripotent stem cell (iPSC)-derived cardiomyocytes (CMs) to model inherited HCM demonstrated in vitro efficacy of mavacamten treatment and concluded that the differences in disease mechanisms related to different genotypes in this population did not change the efficacy of CMIs [59]. These findings show that CMIs could have therapeutic benefit in genotypically diverse HCM populations. Further in vivo studies and clinical trials that are genotype-stratified are required to explore the efficacy of CMIs in diverse genetic patient populations.

## 8. Conclusions

Cardiac myosin inhibitors, namely mavacamten and aficamten, represent a significant advancement in the treatment of HCM, offering a disease-modifying approach that directly addresses the underlying hypercontractility. Clinical trials have demonstrated their efficacy in reducing LVOT obstruction, improving functional capacity, and enhancing quality of life for patients with HCM. In general, the favorable safety profile of CMIs further supports their potential as a promising treatment option for HCM. Research continues to explore the extended applications and effects of these drugs, with possible efficacy in other cardiac conditions. While short-term analyses and use of these drugs seem to be safe and effective, further data from long-term use of these CMIs and use in different populations of patients will provide critical information to determine their role.

## Figures and Tables

**Table 1:** Key differences in the mechanism of action of mavacamten and aficamten.

	Mavacamten	Aficamten
Class of Drug	Cardiac myosin inhibitor	Cardiac myosin inhibitor
Binding Site	Allosteric site distinct from aficamten	Same allosteric site as blebbistatin near pi-releasing backdoor of myosin
Type of Change in Myosin Confirmation	Stabilizes super relaxed state of myosin	Stabilizes weak actin binding pre-power stroke state
Key Mechanism of Action	Decreases the number of myosin heads available to form to crossbridge cycle with actin [22]	Decreases ATPase activity and sarcomere force through slowing phosphate (Pi) release [22]
Effect on Actin–Myosin Relationship	Reduces myosin–actin binding	Prevents the transition to strong actin-binding, force generating
Interaction with Blebbistatin	Does not compete with blebbistatin	Competes with blebbistatin (mutually exclusive binding) [22]
Half-Life and Steady State Confirmation	Long half-life (7–9 days), steady state in ~6 weeks [22]	Shorter half-life, reversible within 24–48 h, steady state in ~2 weeks [22]

**Table 2:** Overview of key completed clinical trials of mavacamten in HCM.

Trial Name	Phase	Population	Method and Design	Primary Endpoints	Key Results/Outcomes
PIONEER-HCM [29]	2	Symptomatic obstructive HCM	Open-label, multicenter, mavacamten as monotherapy in cohort A and add to β-blockers in cohort B	Change in LVOT gradient at 12 weeks	Cohort A: LVOT gradient reduced from 103 mm Hg to 19 mm Hg (mean change: −89.5 mm Hg; *p* = 0.008); Peak VO_2_ increased by 3.5 mL/kg/min; LVEF decreased by 15%. Cohort B: LVOT gradient reduced from 86 mm Hg to 64 mm Hg (mean change: −25.0 mm Hg; *p* = 0.02); Peak VO_2_ increased by 1.7 mL/kg/min; LVEF decreased by 6%
MAVERICK-HCM [30]	2	Symptomatic non-obstructive HCM (NYHA II–III, LVEF ≥ 55%, NT-proBNP ≥ 300 pg/mL)	Randomized, double-blind, placebo controlled with initial 5 mg dose daily with 1 dose titration at week 6	Safety and tolerability with secondary outcomes of NT-proBNP and hs-cTnI	Generally well tolerated and safe with 10% serious adverse effects of mavacamten group and 21% in placebo group. In addition, 5 participants stopped taking the drug due to LVEF reduction which was reversible within 4–12 weeks
EXPLORER-HCM [31]	3	Symptomatic obstructive HCM (NYHA II-III, LVOT gradient above 50 mmHg)	Double-blind RCT with placebo control and 30-week treatment period	Composite of ≥1.5 mL/kg/min increase in peak VO_2_ and ≥1 NYHA class improvement, or ≥3.0 mL/kg/min increase in peak VO_2_ without NYHA class worsening	Improved exercise capacity and symptoms, primary outcomes achieved in 37% mavacamten-treated versus 17% in placebo-treated patients (*p* = 0.0001). Peak VO_2_ increased by 1.4 mL/kg/min (*p* = 0.0006) in mavacamten group with significant improvements in NYHA class and quality of life measures.

Abbreviations: HCM: hypertrophic cardiomyopathy; LVEF: left ventricular ejection fraction LVOT: left ventricular outflow tract; NYHA: New York Heart Association; RCT: randomized controlled trial.

**Table 3:** Overview of key completed clinical trials of aficamten in HCM.

Trial Name	Phase	Population	Method and Design	Primary Endpoints	Key Results/Outcomes
REDWOOD-HCM [33]	2	41 patients with symptomatic nHCM patients with LVOT gradient ≤ 30 mmHg, LVEF ≥ 60%, NT-proBNP > 300 pg/mL	Open-label trial where patients received individualized aficamten doses of 5–15 mg once daily (titration based on echocardiography LVEF) for 10 weeks	Safety, tolerability and efficacy of aficamten (through measures of symptom burden and cardiac biomarkers) over 10 weeks	55% showed NYHA class improvement, 29% became asymptomatic. NT-proBNP reduced by 56% (*p* = 0.001), hs-TnI by 22% (*p* = 0.005). Mean LVEF declined by 5.4%; 3 asymptomatic patients had transient LVEF < 50%. One fatal arrhythmia in a high-risk patient
SEQUOIA-HCM [32]	3	282 patients with symptomatic obstructive HCM, LVEF ≥ 60%, LV wall thickness ≥ 15 mm	Double-blind, randomly assigned to take aficamten (5 mg, starting) or placebo for 24 weeks	Change in peak VO2 from baseline to 24 weeks	Peak Vo2 increased by 1.7 mL/kg/min vs. placebo (*p* = 0.0001). KCCQ-CSS improved by 12 vs. 5 points (*p* < 0.001). 49.3% achieved LVOT < 30 mmHg after Valsalva vs. 3.6% on placebo. Serious adverse events: 5.6% vs. 9.3%

Abbreviations: HCM: hypertrophic cardiomyopathy; KCCQ: Kansas City Cardiomyopathy Questionnaire Clinical Summary Score; LVEF: left ventricular ejection fraction LVOT: left ventricular outflow tract; nHCM: non-obstructive hypertrophic cardiomyopathy; NYHA: New York Heart Association; RCT: randomized controlled trial.

**Table 4:** Overview of most common adverse events of mavacamten and aficamten in HCM.

Adverse Events	Mavacamten (%)	Aficamten (%)
Dizziness	19% ^2^	4.2% ^3^
Atrial Fibrillation	19% ^2^	2.8% ^3^
Death	1.4% ^1^	0% ^1^
LVEF < 50%	10.6% ^1^	4.8% ^1^
Heart Failure	4.3% ^1^	0% ^1^
LVEF Drop with Temporary [or Permanent] Discontinuation	6.8% [1.9%] ^1^	0.5% [0%] ^1^

Abbreviations: HCM: hypertrophic cardiomyopathy; LVEF: left ventricular ejection fraction. ^1^ Cumulative data taken from systematic review of 10 clinical trials of CMIs [41]. ^2^ Data taken from PIONEER-HCM, Pilot Study Evaluating MYK-461 in Subjects with Symptomatic Hypertrophic Cardiomyopathy and Left Ventricular Outflow Tract Obstruction [29]. ^3^ Data taken from SEQUIOA-HCM, Safety, Efficacy, and Quantitative Understanding of Obstruction Impact of Aficamten in HCM [32].

## Data Availability

No new data were created or analyzed in this study. Data sharing is not applicable to this article.

## Abbreviations

The following abbreviations are used in this manuscript:

HCM	hypertrophic cardiomyopathy
CMI	cardiac myosin inhibitors
LVOT	left ventricular outflow tract
oHCM	obstructive hypertrophic cardiomyopathy
nHCM	non-obstructive hypertrophic cardiomyopathy
ATP	adenosine triphosphate
LVEF	left ventricular ejection fraction
NYHA	New York Heart Association
ECG	electrocardiography
KCCQ-CSS	Kansas City Cardiomyopathy Questionnaire Clinical Summary Score
HCMSQ-SoB	Hypertrophic Cardiomyopathy Symptom Questionnaire Shortness-of-Breath sub-score
HFpEF	heart failure with preserved ejection fraction
FDA	Food and Drug Administration
EMA	European Medicines Agency
